# Fiber-assisted nanoparticle tracking analysis meets nanorheology: a novel approach for probing viscoelastic properties at the nanoscale

**DOI:** 10.1515/nanoph-2024-0754

**Published:** 2025-03-24

**Authors:** Torsten Wieduwilt, Hannah Geisler, Ronny Förster, Adrian Lorenz, Markus A. Schmidt

**Affiliations:** Leibniz Institute of Photonic Technology, Jena, Germany; ams OSRAM, Regensburg, Germany; Carl Zeiss AG, Jena, Germany; Otto Schott Institute of Material Research, Friedrich Schiller University Jena, Jena, Germany

**Keywords:** nanorheology, nanoparticle analysis, hollow core fiber

## Abstract

This study introduces fiber-assisted nanoparticle tracking analysis (FaNTA) as a platform for nanorheology that utilizes an advanced antiresonant optical fiber to analyze the viscoelastic properties of fluids at the nanoscale. The platform confines colloidal nanotracers within a fiber-integrated microchannel, significantly extending observation times and improving statistical accuracy. The FaNTA system consists of a custom-designed microstructured antiresonant fiber, a dedicated optical setup, and sophisticated data processing including image analysis and statistical filtering, enabling precise determination of the hydrodynamic diameter and thus the local viscosity. The study demonstrates the capabilities of the FaNTA concept in the context of rheology by measuring the viscosity of glycerol-water solutions at different concentrations using 50 nm gold nanospheres as nanoprobes. By analyzing their individual diffusive motion, the platform accurately determines fluid viscosities with results that closely match literature values, validating the efficacy of FaNTA for nanorheological applications. FaNTA’s high accuracy and performance in nano- and microrheological measurements highlight its broad potential in nanoscale materials science, dynamic process studies, life and environmental sciences, and nanochemistry. This innovative approach provides a valuable extension to current microrheological methods and offers precise nanoscale fluid characterization for a wide range of applications.

## 1 Introduction

Studying the properties of fluids at the nanoscale level is essential for a multitude of fields of research and application [1], [2], including biomedicine (e.g., drug delivery [3]), environmental science (e.g., water purification [4]), and food control research (e.g., stabilize emulsions [5]). In this context, nanorheological approaches are crucial to study the local properties of soft matter using tracer probes such as nanoparticles (NPs) [6]. Here, the field is divided into two main directions: Active approaches, such as those using magnetic forces or optical tweezers, rely on external forces to manipulate NPs in viscoelastic environments, which can have unintended effects on the system [7], [8]. Passive nanorheological techniques, which exploit the thermally activated motion of tracer NPs, have, therefore, become increasingly attractive for studying the viscoelastic properties of complex fluids. Consequently, there is a growing need for innovative approaches to achieve high-precision characterization of NP diffusion using that concept.

One approach enabling precise analysis of individual NPs is nanoparticle tracking analysis (NTA) [9]. NTA works by retrieving the trajectories of individual diffusing NPs using image-based tracking and analyzing the trajectories using Mean-Square-Displacement (MSD) analysis, resulting in the individual diffusion coefficients and finally, through the Einstein–Stokes relation, the hydrodynamic diameters. Since MSD involves statistics, the accuracy of NTA in determining the diffusion coefficient is limited by the length of the trajectories (*N*_f_), with the uncertainty being approximately proportional to $N_{\text{f}}^{-0.5}$ [10], [11]. NTA has been used in nanorheology in a variety of scenarios [12], [13], including studying biological matter for probing the bending mechanics of lipid membranes [14], measuring the microheterogeneity of actin filament networks [15], [16], [17], analyzing DNA [18] or protein [19] solutions, or investigating synthetic polymer systems such as Carbopol [20] or complex fluids in general [21].

One NTA approach recently invented that offers substantially improved statistical accuracy is fiber-assisted NTA (FaNTA) [22]. In this method, NPs diffusing in a liquid-filled microchannel along an optical fiber are illuminated by the modal field and the trajectories are obtained from the sidewise scattered light. In addition to light line illumination and fast readout, the key advantage of FaNTA is its ability to confine NP diffusion within the fluidic channel, keeping the NPs in the field-of-view for extended periods of time: for instance, nanobore optical fibers enable the observation of fast diffusing nano-objects (*D* ≈ 6 μm^2^ s^−1^) over *N*_f_ = 100.000 frames, providing exceptional statistical accuracy [23]. Various FaNTA schemes have been implemented including 3D tracking in modified step-index fibers [24] and NP sizing in antiresonant element fibers [11], achieving characterization of NPs as small as 10 nm [25]. Recently, the technique has been extended to on-chip nanoprinted hollow-core waveguides [26]. As a result, FaNTA is emerging as a versatile platform for advancing nanorheological investigations not previously explored in this context, offering unprecedented capabilities for tracking NPs and analyzing viscoelasticity-related properties.

**Figure 1:**
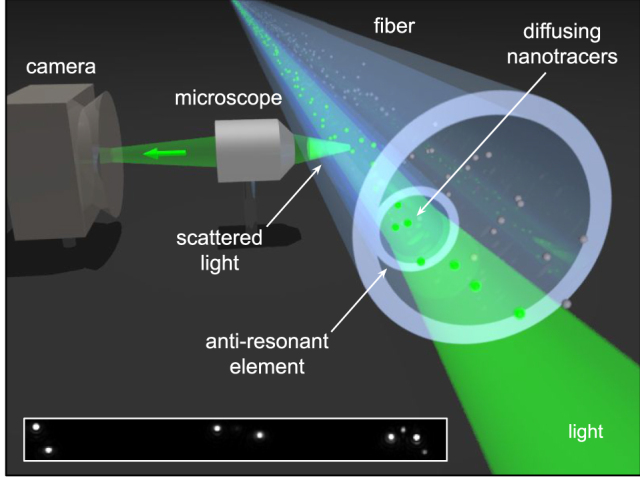
Illustration of the concept of FaNTA-based nanorheology used here to determine the viscoelastic properties of composite liquids (green: guided and scattered light; light blue: antiresonant element fiber; green disks: diffusing nanotracers). The inset shows a microscope image of a (120 × 12) μm section of the fiber microchannel containing eight 50 nm gold nanospheres. Note that the nanotracers are illuminated by the incident light and appear as clearly visible spots in the images, allowing their diffusive motion to be followed.

In this work, we demonstrate that the FaNTA concept, leveraging a sophisticated antiresonant fiber, can effectively probe the viscoelastic properties of selected fluids at the nanoscale level, establishing an innovative platform for nanorheology (Figure 1). Specifically, by studying the Brownian motion of gold NP-based nanotracers within the light-guiding microchannel of a single antiresonant element fiber filled with different glycerol-water solutions, precise determination of the dynamic viscosity of the fluid was achieved. To confirm the accuracy of our results, the viscosities were compared with established literature values from conventional measurement techniques, showing excellent agreement and validating the potential of our approach.

## 2 Concept

### 2.1 Background

The concept discussed in this study involves determining the mean hydrodynamic diameter of a well-characterized ensemble of NPs diffusing in the liquid of interest (here a glycerol-water solution) by FaNTA and MSD analysis. The known mean diameter is then used to calculate the unknown dynamic viscosity of the liquid, using water as a reference.

### 2.2 Mean square displacement analysis (MSD)

The key to the MSD approach is the analysis of the Brownian motion of the NPs, which is a statistical process characterized by the diffusion coefficient *D*. This coefficient can be obtained by analyzing the temporal behavior of the NP, i.e., its position as a function of time *x*(*t*). The resulting MSD is linearly dependent on the time interval Δ*t*, according to the relationship:

(1) $$\text{MSD}\left(i\Delta t\right)=\left\langle {\left[x\left(t+i\Delta t\right)-x\left(t\right)\right]}^{2}\right\rangle =2D\Delta t$$

where *x*(*t*) is the position of the particle at time *t*, Δ*t* is the lag time (time interval between two recorded frames), *i* the lag frame, and ⟨ ⟩ indicates an expectation value. The relationship between the diffusion coefficient and the dynamic viscosity of a medium *η* for a spherical NP of hydrodynamic diameter *d* is given by the well-known Stokes–Einstein equation [9]

(2) $$d\approx \frac{k_{\text{B}}T}{3\pi \eta D}$$

where *T* is the temperature of the medium and *k*_B_ is the Boltzmann’s constant.

As shown in Eq. (2), obtaining accurate values for the viscosity of the glycerol-water solution *η*_s_ requires precise knowledge of the exact diameter of the NP. Due to the manufacturing process, particle solutions have a certain diameter distribution characterized by the mean value *μ* and the coefficient of variation CV = *σ*/*μ* (*σ*: standard deviation). Since the diameter of a single NP tracer is, therefore, not known, *η*_s_ is determined here by calculating the average hydrodynamic diameter of an ensemble of NPs (number of NPs *N*_p_ ≈ 30) and reference it to ultrapure water, where the viscosity is well known. It is important to note that temperature plays a critical role, especially since the dependence of viscosity on temperature becomes more pronounced with increasing glycerol concentration (see Section 5.1 for more details). Accurate temperature control is, therefore, essential for reliable viscosity measurements in such systems. We used a platinum resistance thermometer (PT100) to determine the current temperature, which was attached a few millimeters next to the fiber.

Specifically, the determination of *η*_s_ depends on two critical aspects: (i) the accurate determination of the mean NP diameter and (ii) the precise knowledge of the temperature dependence of the viscosity of water *η*_w_(*T*). As demonstrated in our previous works [27], the first aspect involves complex data processing, including statistical filtering by *z*-scoring (details are available in the Supplementary Information S5) and weighted averaging of the hydrodynamic diameters [11]. The second aspect, the temperature dependence of water viscosity, is well documented and widely reported in the literature. Three types of hydrodynamic diameter obtained from MSD analysis are relevant to the viscosity determination performed in this work:

– the hydrodynamic diameter when the NP is diffusing in water (w): ${\bar{d}}_{\text{w}}=\frac{k_{B}T_{\text{w}}}{3\pi \eta_{\text{w}}\left(T_{\text{w}}\right){\bar{D}}_{\text{w}}}$

– the hydrodynamic diameter when the NP is diffusing in the solution of unknown viscosity (s): ${\bar{d}}_{\text{s}}=\frac{k_{B}T_{\text{s}}}{3\pi \eta_{\text{s}}\left(T_{\text{s}}\right){\bar{D}}_{\text{s}}}$

– the artificial hydrodynamic diameter obtained from solution measurements and assuming that the NP would hypothetically diffuse in water (a): ${\bar{d}}_{\text{a}}=\frac{k_{B}T_{\text{s}}}{3\pi \eta_{\text{w}}\left(T_{\text{s}}\right){\bar{D}}_{\text{s}}}$

Here, *μ*(*T*) is the viscosity of the respective medium at temperature *T*, $\bar{d}$ is the average hydrodynamic diameter (incl. *z*-score filtering and weighted averaging), and $\bar{D}$ is the average diffusion coefficient. Note that ${\bar{d}}_{\text{s}}$ and ${\bar{d}}_{\text{w}}$ contain different diffusion coefficients, temperatures, and viscosities. Assuming that the calculated hydrodynamic diameters of the NPs in water and in solution are identical ${\bar{d}}_{\text{w}}={\bar{d}}_{\text{s}}$ the viscosity of the solution at temperature *T*_s_ is given by:

(3) $$\eta_{\text{s}}\left(T_{\text{s}}\right)=\frac{{\bar{d}}_{\text{a}}\left(T_{\text{s}}\right)}{{\bar{d}}_{\text{w}}\left(T_{\text{w}}\right)}\eta_{\text{w}}\left(T_{\text{s}}\right)$$

It should be noted that the introduction of the artificial diameter ${\bar{d}}_{\text{a}}$ is important because it allows the employment of the *z*-scoring approach to increase the accuracy of the statistical analysis. If only the diffusion constants are considered, the *z*-scoring cannot be used because a Gaussian distribution can only be assumed for the NP diameter distribution and not for the diffusion coefficient.

### 2.3 Waveguide platform

**Figure 2:**
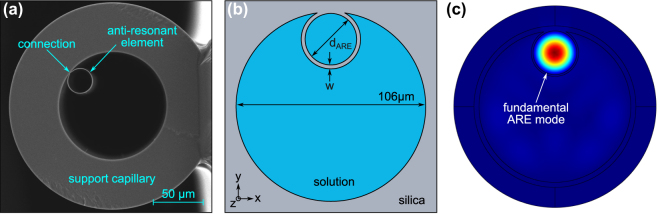
Overview on the single-element antiresonant (1-ARE) fiber used for the FaNTA experiments. (a) Scanning electron microscopy (SEM) image of the fiber cross section, showing the light guiding element and the supporting fiber-type capillary. (b) Corresponding schematic representation including the main geometric parameters (ARE channel diameter and membrane thickness *d*_ARE_ and *w*) and the definition of the coordinate system. The blue area indicates the solution containing the NP tracers, and the gray areas are silica. (c) Electric field distribution (norm of electric field, linear color scale) of the fundamental ARE mode (HE_11_) inside the ARE (*n* = 1.333, *d*_ARE_ = 17.3 μm, *w* = 732 nm, *λ*_0_ = 532 nm). Note that the exact size of the large capillary supporting the ARE plays a minor role for mode formation, its thickness and inner diameter was decreased for faster computation.

The waveguide platform used is a single-element antiresonant hollow-core (1-ARE) fiber (fiber cross section shown in Figure 2(a) and (b)) [11]. The light guiding element is a thin-walled capillary (inner diameter *d*_ARE_) attached to the inner wall of the supporting fiber-like capillary (inner diameter ≈ 106 μm), guiding the light through the antiresonant effect in a fundamental Gaussian-type mode (Figure 2(c)) calculated by Finite-Element modeling). Note that such intensity distribution of the mode is well suited for NTA where the diffusing NPs are directly illuminated by the mode field. An analytical approach to describe light propagation in a 1-ARE fiber is the tubular antiresonant waveguide model [28]. In this model, the glass wall is treated as a thin planar glass membrane where incident and reflected waves of the two interfaces interfere, creating spectral intervals of high (antiresonant) and low (resonant) transmission. Here, the membrane thickness *w* is the key parameter for spectral matching the transmission bands, i.e., the antiresonances (center band wavelengths), which are given by [29]

(4) $$\lambda_{\text{A}}=\frac{4w}{2m-1}\sqrt{n_{\text{m}}^{2}\left(\lambda \right)-n_{\text{l}}^{2}\left(\lambda \right)}$$

with the order of the resonance *m* and the refractive indices of glass membrane *n*_m_ and liquid *n*_l_.

At an operating wavelength of 532 nm, antiresonance is achieved with membrane thicknesses of *w* ≈ 225 nm (*m* = 1), *w* ≈ 675 nm (*m* = 2) and *w* ≈ 1.12 μm (*m* = 3) for water and *w* ≈ 280 nm (*m* = 1), *w* ≈ 860 nm (*m* = 2), and *w* ≈ 1.43 μm (*m* = 3) for 40 wt% glycerol-water solution (see Figure S3a and b in SI-Section 4). Here, we use a lower-order band to avoid expanding the junction (Figure 2(a)) between the capillary wall and the ARE, as this would increase the power dissipation, resulting in transmission losses.

## 3 Methods

### 3.1 Nanoparticle solution preparation

Five suspensions with different glycerol weight concentrations (0 % < *c* < 40 %, Δ*c* = 10 %) were prepared using a 50 nm gold NP stock solution (Cytodyagnostics Inc., *c* = (3.5 ± 0.3)10^10^ NPs/mL, CV < 10 %, *d* = (58 ± 4) nm (measured with Zetasizer)) and anhydrous glycerol (MERCK, more details can be found in Supplementary Information S3). Dilution was necessary due to the high NP concentration in the stock solution. The degree of dilution depended on the observation volume of the selected fiber channel $V_{\text{obs}}=\pi {\left(d_{\text{ARE}}/2\right)}^{2}\times L$ (*L*: length of the observed fiber section), the desired number of particles in the observation volume *N*_p_ ≈ 30), and the amount of glycerol added. Note that the NP concentrations used are a compromise between maximizing the number of trajectories evaluated and ensuring that trajectory crossings are avoided, which can lead to issues with correct NP linking in the image processing.

### 3.2 Optical setup

Light from a 532 nm CW laser (Verdi G, Coherent Corp.) was coupled into a 15 cm section of the 1-ARE fiber via fiber-to-fiber butt coupling using a single-mode, low-NA delivery fiber (NA = 0.05, mode field diameter: 8.5 µm). NP motion was observed with a microscope positioned perpendicular to the fiber axis. To maximize the intensity scattered to the microscope, the transmitted light of the delivery fiber was linearly polarized perpendicular to the optical axis using a manual 2-paddle fiber polarization controller. To control the coupling efficiency and orientation of the ARE channel, a camera and a single objective (*M* = 20, NA = 0.4) were installed at the output side of the fiber, which was rotated by a fiber rotator to align the ARE channel with respect to the microscope. A 10× objective (NA = 0.25) was used to image the diffusing NPs inside the ARE channel, approximately 3 cm from the coupling side. In the current experiments, a laser power of 1.5 mW was used at the output of the launching fiber, which corresponds to a value of ≈1 mW at the tracking detection point. This value is well below the value at which the absorption of the laser power, and consequently the heating of the nanoparticle, influences the particle diffusion. The length of the field of view along the fiber axis was ≈1.4 mm. The scattered light from the gold spheres was imaged using a CMOS camera (Basler acA4096-40um) with a sensor size of (4,096 × 2,168) pixels and a pixel size of (3.45 × 3.45) μm. The fiber was mechanically stabilized on a polished quartz block to minimize vibrations and provide a “dark background.” A thin fused silica coverslip (0.4 mm) was placed on the imaged section, and the space between the fiber and the coverslip was filled with index-matching liquid (refractive index: *n*_D_(20 °C) = 1.458). Further details can be found in S1 and Ref. [25].

### 3.3 Data analysis

#### 3.3.1 Tracking

The image-based tracking of the nanospheres was performed using the Python package *Trackpy*. The corresponding script has been previously applied to NTA experiments in different waveguides and with different NPs, including gold, polystyrene, and biological particles such as viruses and phages [27]. The processing of the data (total number of frames per data set: 6,000) is divided into several steps: (i) preprocessing of the raw images (e.g., background removal), (ii) localization of the NPs by their center of mass in each frame, (iii) linking of the trajectories, and (iv) determining the diffusion coefficient of each NP by MSD analysis of the longitudinal part of the trajectories (along *z*-direction). The reason for using only this component of the trajectory is that free NP diffusion happens only along the longitudinal direction, while along the transverse direction, the particle can only diffuse freely until it reaches the channel wall. For a 50 nm NP diffusing in a cylindrical channel of 18 µm diameter filled with water, the free diffusion time is 4.7 s – assuming the particle starts from the center of the channel (best possible position). Considering that most particles do not start in the middle of the channel and some are faster than others, diffusion is confined along the transverse direction for the given total movie length (in case of water 10.9 s, Table S1 in SI Section 1). Note that the number of detected trajectories exceeds the actual number of NP due to intersecting trajectories or NPs becoming temporarily undetectable due to low scattering intensity, especially when NPs reach the channel wall.

#### 3.3.2 MSD analysis

A preliminary analysis showed a linear relationship between MSD and lag time for the first three lag times in all samples, which is a sufficient number to determine the hydrodynamic diameter. Note that a single lag time value represents a statistical average of the positional differences over the entire trajectory, with the shortest lag time including *N*_f_ − 1 values providing the highest statistical significance. Longer lag times include fewer position values and thus show less statistical significance, making the first few lag times the most relevant for analysis [30].

### 3.4 Accuracy and statistical treatment

#### 3.4.1 Discussion

Since longer trajectories improve statistical accuracy, the trajectory lengths were used as weighting factors when averaging the hydrodynamic NP diameters of each ensemble. This reduces the effect of outliers that typically result from insufficient tracking. A total of 6–7 ensembles were measured per solution, with each ensemble containing new NPs entering the observation area, imposed through capillary flow from the sample reservoir.

#### 3.4.2 Example

**Figure 3:**
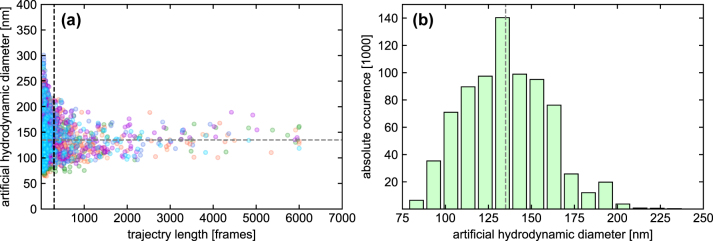
Example of an MSD analysis for the situation of a 30 wt%, glycerol-water solution after *z*-score filtering. (a) Overlay of the artificial hydrodynamic diameter distributions as a function of trajectory length of seven measured ensembles (each color refers to one ensemble). The vertical dotted gray line indicates the limit of 300 frames above which trajectories are considered in the diameter determination procedure. The horizontal dashed lines show the average artificial hydrodynamic diameter $\left({\bar{d}}_{\text{a}}=\left(135\pm 5.1\right)\;nm\right)$ . (b) Corresponding histogram averaged over all seven ensembles (weighted by trajectory length; only trajectories > 300 frames were used). The absolute occurrence corresponds to the sum of all trajectory lengths in the respective bin (bin size = 10 nm). The vertical dashed line indicates the average artificial hydrodynamic diameter (135 nm).

As an example for the characterization of one solution, Figure 3a shows the distribution of the artificial hydrodynamic diameter for seven ensembles measured in a 30 wt% glycerol-water solution. For the calculation of the averaged artificial hydrodynamic diameter, only trajectories longer than 300 frames [11] (marked by the vertical black line) were included, as shorter trajectories yield high statistical uncertainties. To increase the statistical significance of the results, the *z*-score filtering method was used to exclude outliers from the data processing (see SI Section 5 for more details). This parameter is a statistical measure of how many standard deviations a data point is away from the mean and was set here to *z*_max_ = 2.576. In this case, 1 % of the values are further away from the mean than 2.576 times the standard deviation. The final averaged artificial hydrodynamic diameter including all seven ensembles is ${\bar{d}}_{\text{a}}=\left(135\pm 5.1\right)\;nm$ (gray dashed line in Figure 3a, histogramm representation shown in Figure 3b). The standard deviation of the size distribution ${\bar{\sigma }}_{\text{s}}$ is ±23.6 nm resulting in a coefficient of variation $\text{CV}={\bar{\sigma }}_{\text{s}}/{\bar{d}}_{\text{a}}=0.17$ . The CV value calculated from the mean value and the standard deviation of the size distribution for all experiments are in the range 0.16 < CV < 0.2.

#### 3.4.3 Impact of the confinement on diffusion

It is important to consider the effect of the microchannel on diffusion when determining the diameter. In general, confinement affects the diameter determination procedure in two ways:

– A reduction in the MSD, especially at longer observation times, due to the spatial restriction of transverse diffusion.
– An increase in viscosity as the NP approaches the channel wall where fluid flow becomes more restricted.

These effects are specific to transversal particle motion, whereas longitudinal diffusion is only affected by the viscosity increase. Confinement can be accounted for by the hindrance factor *K*_d_, which corrects the diffusion coefficient as *D*_free_ ≈ *D*_confined_/*K*_d_ and depends on the ratio of NP to channel diameter [31], [32]. In the present case (*d*_ARE_ = 18.1 μm and *d*_NP_ = 50 nm), *K*_d_ = 0.983, indicating an error of only ∼1.5 %, which is negligible for this study.

## 4 Results

**Figure 4:**
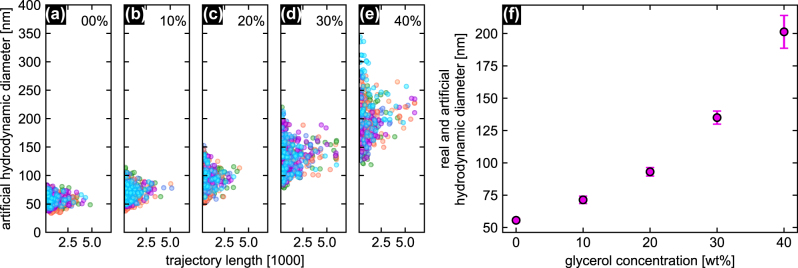
Results of glycerol concentration dependent FaNTA measurements. (a)–(e) Distributions of the calculated NP diameters over the trajectory length for the five different concentrations considered (indicated at the top of each plot). The 6–7 NP ensembles measured for each concentration are superimposed and shown in different colors. (f) Resulting average hydrodynamic diameters for pure water (real hydrodynamic diameter) and glycerol-water solutions (artificial hydrodynamic diameter). The error bars indicate the standard deviations of the mean.

**Figure 5:**
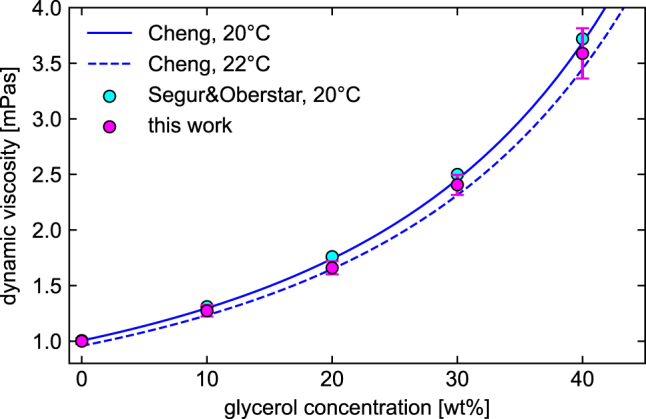
Comparison of dynamic viscosities obtained from the FaNTA experiments performed here (magenta dots, including error bars) with data reported by Segur&Oderstar (magenta dots) and Cheng (blue lines, solid 20 °C, dashed: 22 °C) as a function of glycerol concentration (in wt%).

Comparing the measurement results (i.e., the hydrodynamic diameter/trajectory length dependence) across all five concentrations (Figure 4(a)) shows an increase in the NP diameters with concentration, as expected due to the increasing viscosity of the solution with higher glycerol content. The average artificial hydrodynamic diameters with the respective standard deviations calculated from the size distributions of the individual samples (Figure 4(a)–(e)) are shown in Figure 4(f). To quantitatively evaluate the experimental results, the viscosity of each solution was calculated using (3) and compared with literature values (Figure 5). The comparison is based on empirical formulas derived by Cheng (blue curves) [33] and the data provided by Segur&Oberstar (cyan dots) [34] covering glycerol concentrations from 0 to 100 % and temperatures between 0 and 100 °C. Overall, the FaNTA results (magenta dots in Figure 5) are in excellent agreement with literature data, further confirming the relevance of FaNTA in the context of nanorheology. Note that although not immediately apparent, the values from the work of Cheng et al. are approximately 1–1.8 % lower than the Segur&Oberstar data in the 10 − 40 wt% glycerol range at 20 °C.

## 5 Discussion

Overall, this study is the first to investigate FaNTA in the context of nano- and microrheology, confirming its applicability and relevance through the strong agreement of the results with literature values. Critical parameters and potential future improvements to the current configuration are discussed below.

### 5.1 Influence of the temperature

**Figure 6:**
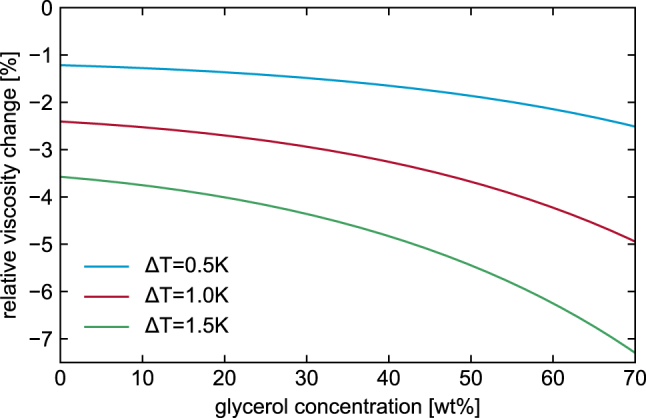
Relative viscosity change of glycerol-water solutions as a function of glycerol concentration for three different temperature deviations (blue: Δ*T* = 0.5 K, red: Δ*T* = 1.0 K, green: Δ*T* = 1.5 K, *T*_0_ = 20 K).

As suggested by Eq. (1), a critical parameter in the entire process is the precise control of temperature. To demonstrate this effect, we have calculated the relative change in dynamic viscosity of the glycerol-water solution Δ*η*_s_(Δ*T*) = (*η*_s_(*c*, *T*_0_ + Δ*T*) − *η*_s_(*c*, *T*_0_))/*η*_s_(*c*, *T*_0_) as a function of glycerol concentration for three temperature deviations Δ*T* (*T*_0_: reference temperature) using the model of Cheng [33] (Figure 6). As shown in Figure 6, the effect of temperature on relative dynamic viscosity becomes more pronounced with increasing glycerol concentration. For example, in a 40 wt% glycerol-water mixture, a change in temperature of 1 K at *T*_0_ = 20 °C results in a 3 % change in relative viscosity. Therefore, measuring higher viscosities generally requires more accurate temperature control.

Another important factor to consider in this context is the heat generated by the nanoparticles and its dissipation into the liquid environment, which can increase temperature in the vicinity of the nanoparticle. This could potentially change the local viscosity and affect the diameter determination. However, based on previous experiments and a theoretical analysis of the heat generated at the nanoparticle surface under different conditions, a temperature-induced change in local viscosity can be safely disregarded (see Supplementary Information, S6).

### 5.2 Improving statistics at high viscosities

Another important observation is that the diameter distribution broadens with increasing viscosity (Figure 4), a trend that is likely to continue at higher viscosities. Consequently, the accuracy for determining diameters decreases with increasing viscosity. Beside using a NP solution with a narrower distribution, it may be beneficial to improve the statistics by measuring more ensembles or increasing the number of NPs per ensemble. However, increasing the NP concentration is limited by the volume of the microchannel, as higher concentrations lead to more particle crossings, which shorten the trajectory length. Conversely, the risk of NP crossings decreases as diffusion slows with increasing viscosity. It is, therefore, important to find the optimum balance between particle concentration and measurement time.

### 5.3 Influence of impurities and agglomerates

For the material used here, complete absence of impurities in the glycerol solution used cannot be guaranteed, as filtration with nanopore filters (<100nm) is challenging due to the high viscosity. In addition, occasional agglomeration of NPs may occur, which should be prevented by the added sodium-citrate, but cannot be excluded. Note that the data processing procedure (e.g., by *z*-scoring filtering) aims to eliminate such outliers.

### 5.4 Comparison to commonly used methods

A widely used method to characterize NPs is dynamic light scattering (DLS) [35]. Here, NTA-based approaches have the advantage of being able to characterize individual NPs, whereas DLS analyzes the temporal variations of the scattered light of the entire NP ensemble. As a result, large NPs can bias the calculated hydrodynamic diameter in DLS due to their large scattering cross section. In FaNTA, the recorded scattering intensity provides an additional degree of freedom to evaluate individual NPs that is not available in DLS. For example, the scattering cross section of a 50 nm polystyrene NP (*n* = 1.56) is 685 times smaller than that of a gold particle of the same size and can, therefore, be excluded from the data set. Note that a 160 nm diameter polystyrene NP is required to achieve an identical scattering cross section. Such large NPs are effectively removed by the *z*-score filtering used here. Therefore, the FaNTA-based approach discussed in this work uniquely enables the separation of colloidal nanotracers from impurities and other interfering particles, improving performance for nanorheological characterization.

Among available techniques, video particle tracking approaches, such as NTA, provides an optimal balance of spatial and temporal resolution, versatility, and ease of implementation for nanorheology studies of soft matter and biopolymer solutions, as it overcomes limitations of diffusing wave spectroscopy and avoids the complexities and limitations associated with optical and magnetic tweezers (see Supplementary Information S7 for a detailed comparison).

### 5.5 Influence of surface functionalization

The gold NPs used in this study are reactant-free, with no surface functionalization to prevent interactions between functional groups and the analyte. They are coated only with a thin layer of sodium citrate, which imparts a negative charge to prevent agglomeration. This choice is important because the chemical properties, ionic character, and ionic strength of the surrounding medium influence the electrochemical double layer around the particle, which affects the hydrodynamic diameter. Gold NPs with other coatings, such as a polyethylene glycol (PEG) chain ending in a carboxyl group (e.g., from NanoComposix), may introduce potential interactions, as the glycerol OH groups could chemically interact with the PEG carboxyl group, potentially altering the hydrodynamic diameter. Therefore, NP solutions without surface functionalization are preferred, or confirmation of any chemical interactions with the surrounding medium is recommended.

### 5.6 Comparison to other approaches

FaNTA offers certain advantages over other microrheological methods and conventional rheology. While standard viscometers (e.g., Ostwald, Höppler, rotational viscometers) are generally more suitable for high viscosity samples and require larger volumes, fiber-based environments are ideal for studying low viscosity ranges, tiny sample volumes, and local viscosity inhomogeneities. Other NTA implementations are often limited by particle velocity due to low camera readout speeds. In contrast, FaNTA offers a viable alternative as the small image size allows for high acquisition speeds – up to 700 fps can easily be achieved.

### 5.7 Improvement of fiber design

A key element of the FaNTA scheme used here is the complex microstructured optical fiber that guides the light in a selected microchannel [28]. While the presented fiber design is state-of-the-art, the flexibility of fiber drawing allows further advances, such as tailoring NP illumination through modal engineering (e.g., flat field formation [36]) or integration with microfluidics (e.g., as demonstrated here [37]). The latter is particularly feasible due to the comparatively large core diameter, which allows rapid fluid exchange.

### 5.8 New implementation approaches

In addition to optical fibers, recent advances in 3D nanoprinting technology have enabled the transfer of the light guiding concept from fiber optics to planar waveguide technology, effectively opening up the field of on-chip hollow core waveguides for NTA research. Promising approaches such as locally structured light cages [26] and microgap waveguides [38] have recently been demonstrated. Here, the direct coupling of nanoprinted waveguides with delivery fibers offers a way to achieve a higher degree of photonic and microfluidic integration, as exemplified by fiber-interfaced light cages [39].

### 5.9 Viscosity range

Due to the inverse proportionality between diffusion coefficient and viscosity, higher viscosities result in lower diffusion coefficients. For example, for the same NP diameter, the diffusion coefficient in pure glycerin is approximately 1,400 times lower than in pure water, requiring lower frame rates and longer acquisition time. One solution to compensate for this effect is to use smaller NPs. Here, a NP diameter of about 16 nm in a liquid with a viscosity of ∼100 mPa⋅s gives the same diffusion coefficient as a 50 nm NP in water. Note that the FaNTA approach allows to characterize gold NPs diffusing in water down to a diameter of 10 nm [25]. Another important factor is the filling time of the ARE channel, which is described by the Lucas–Washburn equation [40]. Assuming water (*η* = 1 mPa⋅s), a channel diameter of 18 µm and a filling length of 5 cm, the filling time is about 10 s. However, for a viscosity of 10 mPa⋅s (∼60 wt% glycerol-water solution) or 100 mPa⋅s (∼85 wt% glycerol-water solution), the filling time increases to approximately 2 min and 18 min, respectively. Thus in case high viscosities are considered, it is, therefore, advisable to apply vacuum during the filling process.

### 5.10 Future rheological experiments relying on elastic light scattering

The specific properties of elastic light scattering are critical in FaNTA and offer significant potential for nanorheology research. For example, rotational diffusion of asymmetric nano-objects such as gold nanorods can be revealed by analyzing the temporal variations of the elastically scattered light [41], [42], [43]. As rotational diffusion is significantly faster than translational diffusion, the characterization of rotational diffusion by FaNTA could be valuable for the study of fast viscoelastic processes. Furthermore, as a coherent process, elastic light scattering allows interference between light scattered by neighboring nanoparticles, providing nanoscale distance information, as previously shown for FaNTA [22]. This approach is particularly relevant to nanorheology, where viscosity changes as nanoparticles approach each other at nanoscale levels. Similarly, interference of scattered light from tracer nanoparticles and the channel wall can provide information on nanoparticle-wall distances, facilitating studies of viscoelastic boundary layer effects, including the no-slip condition [44], under varying fluid environments.

### 5.11 Application in rheology and beyond

As demonstrated in this work, the characterization of nanoscale viscoelastic properties is a key application of FaNTA [45], [46], and examples of application include understanding soft matter [15], characterizing nanoscale inhomogeneities [47], and exploring phase transitions [48]. In addition to understanding static properties, FaNTA enables visualization of dynamic processes such as gelation (e.g., thermosensitive gel formation [49]), polymerization (e.g., reversible addition-fragmentation chain transfer polymerization [50]), or material degradation [51]). In addition, due to the presence of the microchannel sidewalls, FaNTA can be used to study nanoscale liquid behavior near surfaces, where effects are different from bulk behavior (see hindrance factor). Here, the flexibility in fiber design allows these effects to be enhanced and studied at high resolution using smaller microchannels. Relevant FaNTA-related studies in this context are the three-dimensional tracking of nanoparticles in modified step-index fibers [24] or flat-field formation in nanobore optical fibers [36]. Due to its ability to characterize NPs with high precision, FaNTA has a wide range of applications even beyond rheology, including environmental science (e.g., detection of pollutants in water) [52], [53], medical research (e.g., characterization of vesicles [54], [55] or study of protein–protein interactions [56]), quality control of synthetic NPs (e.g., characterization of synthetic NPs [57]), and understanding of nanoscale processes (e.g., analysis of thermo-responsive or solvency-sensitive core-satellite nanoassemblies [26], [58]).

## 6 Summary and outlook

This study presents the first demonstration of the FaNTA platform in the context of nanorheology, using a custom-designed antiresonant fiber and diffusing nanotracers to probe the viscoelastic properties of selected fluids. A key feature enabling this study is the confinement of diffusing colloidal nanotracers within a fiber-integrated microchannel, which allows for exceptionally long observation times and thus excellent statistical significance. The experimental setup, which includes a single-element antiresonant fiber, a dedicated optical setup, and advanced data processing, allows precise determination of hydrodynamic diameters while taking into account critical experimental factors such as temperature. The ability of FaNTA to measure nanoscale viscosity was validated by tracking 50 nm gold nanospheres in glycerol-water solutions of various concentration ratios, showing excellent agreement with literature values. This work demonstrates the potential of FaNTA and highlights its high performance in nano- and microrheological applications, suggesting its versatility in nanoscale research, materials science, life sciences, and nanochemistry. In addition, the fiber-based nanorheology platform opens up possibilities for photonic integration with fiber and planar photonic circuits, establishing FaNTA as a valuable extension to current nanorheology techniques for precise nanoscale fluid characterization.

## Supplementary Material

Supplementary Material Details
