# Supplementary material for: Fiber-assisted nanoparticle tracking analysis meets nanorheology: a novel approach for probing viscoelastic properties at the nanoscale
Source: Nanophotonics. 2025 Mar 24;14(7):935–45. doi: 10.1515/nanoph-2024-0754 (PMC11980876; doi:10.1515/nanoph-2024-0754)
Supplement: Supplementary file 1 — Supplementary Material Details [file j_nanoph-2024-0754_suppl_001.pdf]

Supplementary Information

Fiber-Assisted Nanoparticle Tracking Analysis meets Nanorheology: A Novel Approach for Probing Viscoelastic Properties at the Nanoscale

Torsten Wieduwilt[1,**], Hannah Geisler[2,**], Ronny Förster[3], Adrian Lorenz[1] and Markus A. Schmidt[1,4*]

[1]Leibniz Institute of Photonic Technology, Jena, Germany; [2]ams OSRAM, Regensburg, Germany; [3]Carl Zeiss AG, Jena, Germany; [4]Otto Schott Institute of Material Research, Friedrich Schiller University Jena, Jena, Germany
*markus-alexander.schmidt@uni-jena.de
**these authors contributed equally to this work

Sec. 1 Experimental setup

A schematic of the experimental setup is shown in Fig. S1. Light from a 532 nm continuous wave (CW) laser source (Verdi G, Coherent Corp.) is coupled into the measurement fiber (1-ARE) via a single-mode fiber (SMF). A polarization controller produces linearly polarized light perpendicular to the optical axis at the SMF output. The nanoparticle suspension is introduced into the measurement fiber at the light coupling point (butt coupling position). The hollow channel of the fiber containing the diffusing nanoparticles is imaged by a camera mounted on a microscope. A fiber rotator is used to adjust the fiber orientation, while an additional objective and camera at the rear end of the 1-ARE fiber monitor both the orientation and the output mode. To facilitate the flow of particle ensembles into the camera's field of view, a heat source (soldering iron) is placed at the exit of the sensing fiber to vaporize a small amount of liquid, imposing liquid flow. Heating is continued until a uniform distribution of particles is visible in the channel. After each new ensemble is introduced, the fiber end is sealed with gel to prevent mechanical capillary drift.

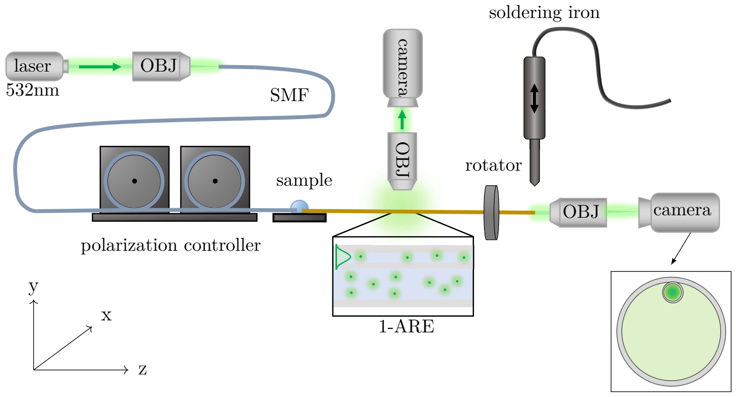

Fig. S1: Schematic drawing of the experimental setup that effectively combines the polarization-controlled light source, the fiber containing the nanoparticle-doped solution of unknown viscosity sample and a microscope with camera for tracking the particle motion.

To achieve a stable position of the fiber without vibration and to obtain a "dark background", the fiber is placed on a polished quartz block, which is located on a xyz-translation stage. Fig. S2 shows a schematic of the sample holder and coverslip.

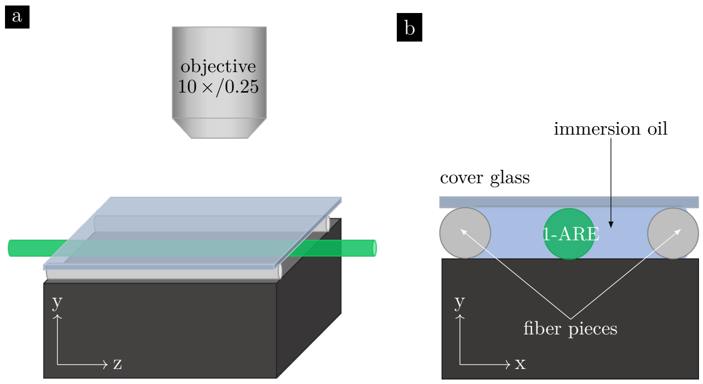

Fig. S2: Sketches of the fiber holder and the arrangement of the 1-ARE fiber. (a) Side view of the configuration showing the position of the microscope objective relative to the measurement fiber. (b) Front view of the observation site: Two fiber pieces positioned alongside the measurement fiber hold the index matching gel in place between the coverslip and the silica block.

Sec. 2 Data acquisition

To achieve high resolution particle tracking, a relatively high frame rate is generally recommended. This approach minimizes particle motion between consecutive frames, reducing the risk of mislinking of nearby objects, which is especially crucial for ensemble studies. However, the frame rate cannot be set arbitrarily high due to the limited shutter and readout speeds of the camera. For tracking experiments, the maximum frame rate should be selected to ensure that particle position shifts can be resolved in successive frames. If no position change is detected during data analysis, the object may be incorrectly classified as stationary. With these limitations in mind, a frame rate of 550 fps was chosen for tracking in water. Since particles diffuse less in more viscous fluids, the frame rate was reduced according to fluid viscosities reported in the literature. The final values are shown in Tab. S1, along with the time required to acquire 6000 frames. This number of frames was chosen for all measurements to ensure reasonable trajectory lengths, allowing the nanoparticles in each sample to travel a similar distance during image acquisition.

Table S1: Parameters used for the image acquisition for all samples (wt% glycerol).

	**water**	**10 wt%**	**20 wt%**	**30 wt%**	**40 wt%**
**framerate [fps]**	550	450	350	250	150
**aquisition time [s]**	10.9	13.3	17.1	24	40

For the experiments presented, an exposure time of 1 ms was chosen for all samples, which resulted in good visibility of the particles during the experiments.

Sec. 3 Preparation of liquid solutions

The preparation of the solutions consists of two main steps:

1. **Dilution of the gold particle stock solution:** A 2 mM trisodium citrate dihydrate water solution was used for dilution, which was filtered through a 20 nm filter (Whatman Anotop syringe filter) to remove unwanted particles before being added to the particle solution. The citrate acts as a stabilizing agent for the particles in the diluted medium

2. **Addition of glycerol for each sample:** After preparing the diluted particle suspension for each sample, the appropriate amounts of glycerol and particle solution were weighed using a balance with an accuracy of 0.1 mg. All components were thoroughly mixed by shaking the tube.

Sec. 4 1-ARE Fiber

Fig. S3 shows the membrane thickness for the first three resonator orders ($m = 1, 2, 3$) as function of the antiresonance wavelength for pure water and 40 wt% glycerol-water solution as ambient medium. For the calculation the refractive index dispersion of fused silica [1], pure water [2] and 40 wt% glycerol-water solution [3] at 20 °C was used.

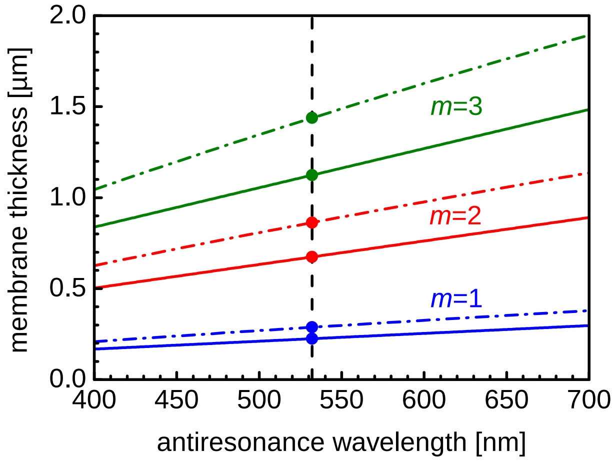

Fig. S3: Membrane thickness for the first three resonator orders ($m = 1, 2, 3$) as function of the antiresonance wavelength (water: solid lines, 40 wt% glycerol-water solution: dash dotted lines). The vertical dashed black line indicates the operation wavelength of 532 nm. The optimal wall thicknesses can be read from the intersection points (marked with the colored dots).

At a operation wavelength of 532 nm, the maximum transmission is achieved at a membrane thickness of $w \approx 225$ nm ($m = 1$), $w \approx 675$ nm ($m = 2$) and $w \approx 1.12$ µm ($m = 3$) for water and $w \approx 280$ nm ($m = 1$), $w \approx 860$ nm ($m = 2$) and $w \approx 1.43$ µm ($m = 3$) for 40 wt% glycerol-water solution.
As already mentioned above, in addition to the wall thickness, the capillary diameter has an influence on the transmission of the light guided in the capillary [4]. Fig. S4 shows the influence of the capillary diameter on the transmission (calculated as loss in dB/cm) of the guided light using the example of water and 40 wt% glycerol-water solution as

the ambient medium. As can be seen in Fig. S4, the losses decrease significantly as the capillary diameter increases. It should be noted that the losses are inversely proportional to the 4th power of the radius of the capillary.

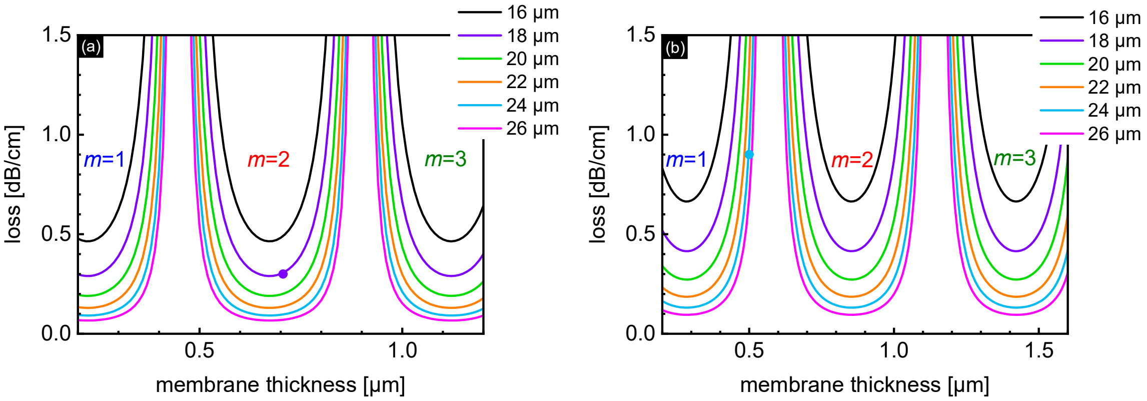

Fig. S4: Loss of the fundamental HE_{11} mode in a single-element antiresonant hollow-core fiber at the wavelength of 532 nm, as a function of the membrane thickness (for the first three resonator orders ($m = 1, 2, 3$)) and capillary diameter ($16 - 26$ µm) for a) water ($n_l = 1.335$) and b) 40 wt% glycerol-water solution ($n_l = 1.385$) as the ambient medium. The refractive index of the membrane was set to $n_m = 1.461$. The fiber dimensions selected for the experiments are marked with a colored dot.

In addition, the range of membrane thickness in which the transmission is maximum is increased, which is advantageous with regard to the accuracy of the fiber production. From the perspective of depth of field, however, there are limits to increasing the channel diameter. It can also be seen that the rise in transmission becomes smaller with increasing capillary diameter. Ultimately, there will be a compromise between depth of field and transmission. In addition, the complexity of the manufacturing process makes it difficult to achieve precise fiber dimensions.

Taking the above facts into account, we selected a capillary with a membrane thickness of $w \approx 700$ nm and a capillary diameter of d_{ARE}=18.1 µm for the experiments with water and a capillary with a membrane thickness of 500 nm and the capillary diameter of 24.3 µm for the experiment with 40 wt% glycerol-water solution (marked with a colored dot in Fig. S4). As can be seen, the selected ARE dimensions for the glycerol solution do not correspond to the optimum values (in terms of maximum transmission). A fiber with optimal geometric parameters was not available at the time of the experiment, so a fiber with near-optimal parameters was used. The resulting attenuation of 1 dB/cm was acceptable given the short fiber length. Tab. S2 provides an overview of the fiber dimensions used in this work.

Table S2: Dimensions of the ARE-channel of the used fibers.

	**water**	**10 wt%**	**20 wt%**	**30 wt%**	**40 wt%**
**SEM wall thickness [nm]**	706	692	718	499	500
**SEM diameter [µm]**	18.1	18.3	18.7	23.1	24.3

Sec. 5 Definition of z-score

To increase the statistical significance of our results, we applied the z-score method to the distribution of hydrodynamic diameters. The z-score quantifies how many standard deviations a data point is from the mean of the data set. For each data point x_i the z-score is calculated as

$$z_\mathrm{i} = \frac{x_\mathrm{i} - \mu}{\sigma_\mathrm{i}} \tag{1}$$

where x_i is the data point, μ is the mean and σ_i is the standard deviation. The advantages of using the z-score include

- **Increased accuracy:** By removing outliers, extreme values that can distort the mean and standard deviation are removed.
- **Improved reliability:** The refined data set more accurately reflects NP diameters, leading to greater statistical confidence.
- **Improved consistency:** The z-score method provides a systematic way to identify and eliminate outliers, ensuring consistency in data analysis.

The z-score process used in MSD analysis discussed here involved several steps:

1. **MSD calculation:** The MSD was determined for each NP by tracking its Brownian motion over time.
2. **Diameter distribution:** The hydrodynamic diameter of each gold NP was derived from the MSD data using the Stokes-Einstein equation to produce a distribution for the NP ensemble.
3. **Outlier filtering:** The z-score method was used to ensure the accuracy of the diameter distribution:
   (a) **Step 1:** The mean and standard deviation of the diameter distribution of particle diameter with more similar trajectory length (trajectory interval processing) were calculated.
   (b) **Step 2:** The z-score was calculated for each diameter.
   (c) **Step 3:** Outliers were identified and excluded if their z-scores exceeded a certain threshold value, which is set here to ± 2.576.

This approach resulted in refined data sets, improving the accuracy and statistical relevance of the nanoparticle diameter distributions.
Fig. S5 shows the influence of the z-score filtering using the example of the 30 wt% glycerol-water solution (all seven ensemble data are shown). The points marked in red are the data that were removed by filtering for further analysis. These data do not fit into the uniform particle hypothesis - have no diameter within a Gaussian distribution. In order to show that short trajectories in particular do not fulfill the Gaussian distribution condition, z-score filtering was applied to all trajectories with a length of more than 50 frames.
For the sample with 30 wt% glycerol-water mixture, $\approx$ 13 % of the trajectories from all seven ensembles were removed from the data set by applying the z-score method. If, as already mentioned in Section 3.4.2 (s. Fig. S3), only trajectories with a length $>$ 300 frames are used, then the proportion of the removed trajectories is lower.

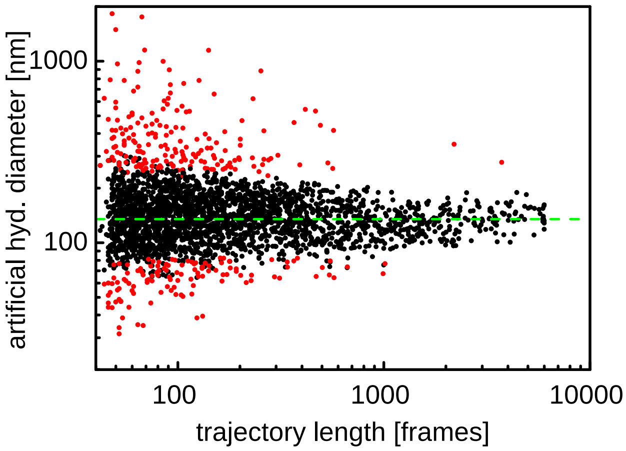

Fig. S5: Result of z-score filtering (black dots) using the example of the 30 wt% glycerol-water solution (all seven ensemble data are shown). The points marked in red are the data that have been filtered out. These data have no diameter within a Gaussian distribution - do not meet the uniform particle hypothesis. The green dashed line indicates the average artificial hydrodynamic diameter (135 nm). In order to make the entire diameter range more visible, logarithmic scaling was used for the diameter in comparison to Fig. S3.

Sec. 6 Impact of nanoparticle heating

The absorbed power generates heat in the nanoparticle, which dissipates into the surrounding liquid, raising the local temperature of the liquid. This increase in temperature can reduce the viscosity near the nanoparticle, leading to a larger diffusion coefficient and consequently a smaller determined hydrodynamic diameter. This phenomenon has been addressed experimentally in a previous publication where the smallest detectable diameter of a gold nanosphere was investigated using the current FaNTA configuration (see Sec. S7 of the Supplementary Information of ref. [5]). In this study, the laser power coupled into the ARE was varied, and tracking experiments were performed with a 50 nm nm gold particle solution (nanoComposix) in ultrapure water containing 2 mM Na-citrate. The experiments showed, that the hydrodynamic diameter decreases when the laser power at the tracking detection location (≈ 3 cm from the fiber entrance) exceeds about 6 mW. Considering the attenuation of the ARHCF (≈ 0.4 dB/cm) and the coupling losses between the launching fiber and the ARHCF (≈ 0.5 dB), this corresponds to a laser power of 9 mW at the output of the launching fiber (butt coupling point). In the FaNTA experiments presented in this manuscript, this effect was taken into account in the experimental design by setting the laser power at the tracking location to ≈ 1 mW, which is well below the threshold for thermal effects impacting nanoparticle diffusion.

Estimation of the temperature generated at the surface of a gold nanoparticle: For a spherical gold nanoparticle, the heat generation by light absorption inside the particle can be calculated by the product of the absorption cross-section σ_{abs} of the particle and the intensity I of the exciting light (in this case, the laser light propagating in the ARE). The temperature increase at the particle surface (steady state solution) resulting from the heat generation is then calculated as follows [6]:

$$\Delta T = \frac{\sigma_{\mathrm{abs}} I}{4\pi k R_{\mathrm{Np}}} \tag{2}$$

where R_{Np} is the nanoparticle radius and k is the thermal conductivity of the surrounding medium. For water, the thermal conductivity is $\approx 0.6\,\mathrm{W/mK}$ (20 °C) [7]. In the case of the glycerol-water solutions, the thermal conductivity is slightly lower (pure glycerol: $k \approx 0.29\,\mathrm{W/mK}$ (20 °C) [8] resulting in a slightly higher temperature at the surface of the gold nanoparticles. The following plot S6 shows the temperature increase for a single 50 nm gold nanoparticle as a function of the laser power (at the tracking detection location) for a pure water environment (black curve) and 40 wt% glycerol-water solution (red curves). The calculations were carried out for light propagation in an ARE with a diameter of 18.1 µm (water and 40 wt% glycerol-water solution) and 24.3 µm (40 wt% glycerol-water solution) using a wavelength of 532 nm.

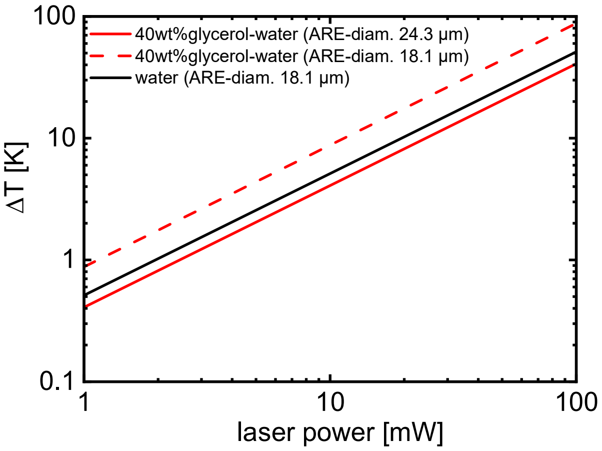

Fig. S6: Calculated temperature increase at the surface of a single 50 nm Au nanoparticle in water (ARE-diameter: 18.1 µm) and 40 wt% glycerol-water solution (ARE-diameter: 18.1 µm and 24.3 µm) as a function of illumination power at the tracking location using a wavelength of 532 nm.

It was assumed that only particles in the center of the ARE (point of greatest laser light intensity) are considered, which overall represents a worst-case scenario. The laser intensity in the center of the corresponding ARE was calculated using the software COMSOL Multiphysics.
The absorption cross-sections of the nanoparticles was calculated using the OMLC Web-based Calculator for Mie Scattering [9]. For this we used the complex refractive index for gold of $n_{\mathrm{Au}} = 0.54 + i2.23$ at $\lambda = 532\,\mathrm{nm}$ [10] and the corresponding refractive indices of the surrounding liquids. All values used for the calculations are listed in Tab. S3

Table S3: Values used for the calculation of the induced temperature increase at the particle surface (at $T = 20\,°\mathrm{C}$ and $\lambda = 532\,\mathrm{nm}$).

	**ARE-diam.** [µm]	$\boldsymbol{n}$	$\boldsymbol{k}$ [W/mK]	$\boldsymbol{\sigma_{\mathrm{abs}}}$ [nm²]	$\boldsymbol{I_{\mathrm{center}}}$ [W/µm²] $P = 1\,\mathrm{mW}$
**pure water**	18.1	1.335	0.60	6770	$1.43 \cdot 10^{-5}$
**40 wt% glyc.-water sol.**	18.1	1.385	0.45	7540	$1.64 \cdot 10^{-5}$
**40 wt% glyc.-water sol.**	24.3	1.385	0.45	7540	$7.65 \cdot 10^{-6}$

As can be seen in Fig. S6, a laser power of 1 mW produces an approximate temperature

increase of about 0.5 K at the surface of a 50 nm gold nanoparticle in water. For a particle in a 40 wt% glycerol-water solution, the temperature increase is slightly lower (0.4 K) because the laser intensity in the centre of the ARE is lower due to the larger ARE-diameter (24.3 µm). Note that for a given channel diameter, the temperature increase is slightly higher than for water due to the higher refractive index and lower thermal conductivity of the liquid. This is illustrated by the dashed red line in Fig. S6, which refers to the situation of the glycerol-water solution for an ARE diameter of 18.1 µm. Note that for all particles outside the center, the temperature increase is significantly lower due to the reduced intensity resulting from the Gaussian-mode type field. Since the laser power used in the experiments is maximal 1 mW, a practically relevant temperature increase of the nanoparticle can be safely ignored.

Sec. 7 Methods Used in Nanorheology

Several major techniques used in the context of nano- and microrheology are shown in Tab. S4, which highlights the advantages, limitations, and typical applications of each method, allowing a direct comparison of their suitability for different experimental conditions.

Table S4: Comparison of techniques used in the context of nanorheology.

**Technique**	**Benefits**	**Limitations**	**Examples, Application**	**Ref.**
**Dynamic Light Scattering (DLS)**	commonly available equipment	cannot resolve spatial heterogeneity, requires high light transmission	polymer gels, biological fluids	[11]
**Diffusing Wave Spectroscopy (DWS)**	probes opaque samples, broad frequency range	cannot resolve spatial heterogeneity	semiflexible polymer networks, actin solutions	[12]
**Video Particle Tracking (VPT)**	allows multiple particle tracking and single-particle tracking for heterogeneity analysis	limited to soft materials, not applicable to stiff materials and non-equilibrium systems	biopolymer solutions, cross-linked network solutions	[13], this work
**Optical Tweezers (OT)**	enables passive and active microrheology, can manipulate objects from 10 µm to 100 nm	requires stiffness calibration of laser traps	polyacrylamide, synthetic and biological gels	[14]
**Magnetic Tweezers (MT)**	produces biologically safe magnetic force fields and gradients to manipulate probe particles	requires the use of magnetic probes	biological networks, cross-linked gels	[15]

Among the techniques listed, video particle tracking (VPT) emerges as a highly suitable approach for nanorheology in soft matter and biopolymer solutions. Unlike dynamic light scattering (DLS) and diffusing wave spectroscopy (DWS), which have problems in resolving spatial heterogeneity, VPT provides both ensemble and single particle tracking

capabilities, allowing characterization of heterogeneous microenvironments. While optical tweezers (OT) and magnetic tweezers (MT) enable active microrheology with precise force application, they require complex instrumentation and calibration procedures that can limit their applicability. In addition, OT is limited by laser-induced heating effects due to the high optical power required for optical trapping, while MT requires the use of magnetic probes that may not be compatible with all sample types. Here, VPT represents an optimal balance between resolution, versatility and implementation effort, making it ideal for the study of soft matter systems such as cross-linked networks and biopolymer solutions. The results of this study therefore indicate that FaNTA, as a VPT method, achieves high statistical accuracy and extended trajectory lengths, surpassing conventional passive microrheology techniques. Based on this comparative analysis, the unique advantages of VPT are justified as a preferred method for nanorheological studies, thus reinforcing the novelty of this approach.

References

[1] I. H. Malitson, “Interspecimen Comparison of the Refractive Index of Fused Silica,” *J. Opt. Soc. Am.*, vol. 55, no. 10, pp. 1205-1209, 1965.

[2] M. Daimon and A. Masumura, "Measurement of the refractive index of distilled water from the near-infrared region to the ultraviolet region," *Appl. Opt.*, vol. 46, no. 18, pp. 3811-3820, 2007.

[3] V. D. Genin, E. A. Genina, V. V. Tuchin and A. N. Bashkatov, "Glycerol effects on optical, weight and geometrical properties of skin tissue," *J. Innov. Opt. Health Sci.*, vol. 14, no. 5, pp. 2142006, 2021.

[4] M. Zeisberger and M. A. Schmidt, "Analytic model for the complex effective index of the leaky modes of tube-type anti-resonant hollow core fibers," *Sci. Rep.* vol. 7, no.1, pp. 11761, 2017

[5] T. Wieduwilt, R. Förster, M. Nissen, J. Kobelke, M. A. Schmidt, "Characterization of diffusing sub-10 nm nano-objects using single anti-resonant element optical fibers," *Nat. Commun.*, vol. 14, no. 1, pp. 3247, 2023

[6] Z. Qin and J. C. Bischof, "Thermophysical and biological responses of gold nanoparticle laser heating", *Chem. Soc. Rev.*, vol. 41, no. 3, pp. 1191–1217, 2012.

[7] The Engineering ToolBox, Water-Thermal Conductivity vs. Temperature, online available at: https://www.engineeringtoolbox.com

[8] O. K. Bates, "Binary Mixtures of Water and Glycerol - Thermal Conductivity of Liquids", *Industrial and Engineering Chemistry*, vol. 28, no. 4, pp. 494-498, 1936

[9] `https://www.omlc.org/calc/mie_calc.html`

[10] P. B. Johnson and R. W. Christy, "Optical Constants of the Noble Metals", *Phys. Rev.*, vol. 6, no. 12, pp. 4370-4379, 1972

[11] B. A. Krajina, C. Tropini, A. Zhu, P. DiGiacomo, J. L. Sonnenburg, S. C. Heilshorn, A. J. Spakowitz, "Dynamic Light Scattering Microrheology Reveals Multiscale Viscoelasticity of Polymer Gels and Precious Biological Materials", *ACS Cent. Sci.*, vol. 3, no. 12, pp. 1294-1303, 2017

[12] J. Y. Huh and E. M. Furst, "Colloid Dynamics in Semiflexible Polymer Solutions", *Phys. Rev. E* vol. 74, no. 3, pp. 031802, 2006

[13] Q. Li, X. Peng, D. Chen and G. B. McKenna, "The Laplace Approach in Microrheology", *Soft matter*, vol. 16, no. 4, pp. 3378–3383, 2020

[14] D. Preece, R. Warren, R. M. L. Evans, G. M. Gibson, M. J. Padgett, J. M. Cooper, M. Tassieri "Optical Tweezers: Wideband Microrheology", *J. Opt.*, vol. 13, no. 4, pp. 044022, 2011

[15] R. M. Evans, M. Tassieri, D. Auhl and T. A. Waigh, "Direct Conversion of Rheological Compliance Measurements into Storage and Loss Moduli". *Phys. Rev. E*, vol. 80, no. 1, pp. 012501, 2009
